# The Pleiotropic Role of L1CAM in Tumor Vasculature

**DOI:** 10.3390/ijms18020254

**Published:** 2017-01-26

**Authors:** Francesca Angiolini, Ugo Cavallaro

**Affiliations:** Unit of Gynecological Oncology Research, European Institute of Oncology, Via G. Ripamonti 435, I-20141 Milan, Italy; francesca.angiolini@ieo.it

**Keywords:** L1CAM, tumor angiogenesis, vascular normalization, antiangiogenic therapy

## Abstract

Angiogenesis, the formation of new vessels, is a key step in the development, invasion, and dissemination of solid tumors and, therefore, represents a viable target in the context of antitumor therapy. Indeed, antiangiogenic approaches have given promising results in preclinical models and entered the clinical practice. However, in patients, the results obtained so far with antiangiogenic drugs have not completely fulfilled expectations, especially because their effect has been transient with tumors developing resistance and evasion mechanisms. A better understanding of the mechanisms that underlie tumor vascularization and the functional regulation of cancer vessels is a prerequisite for the development of novel and alternative antiangiogenic treatments. The L1 cell adhesion molecule (L1CAM), a cell surface glycoprotein previously implicated in the development and plasticity of the nervous system, is aberrantly expressed in the vasculature of various cancer types. L1CAM plays multiple pro-angiogenic roles in the endothelial cells of tumor-associated vessels, thus emerging as a potential therapeutic target. In addition, L1CAM prevents the maturation of cancer vasculature and its inhibition promotes vessel normalization, a process that is thought to improve the therapeutic response of tumors to cytotoxic drugs. We here provide an overview on tumor angiogenesis and antiangiogenic therapies and summarize the current knowledge on the biological role of L1CAM in cancer vasculature. Finally, we highlight the clinical implications of targeting L1CAM as a novel antiangiogenic and vessel-normalizing approach.

## 1. Tumor Angiogenesis

Solid tumors are unable to grow beyond a certain size (usually ~2 mm^3^) in the absence of blood vessels that supply oxygen and nutrients to cancer cells and remove the metabolic waste. Blood vessels, in addition, provide cancer cells with a route to enter the circulation and eventually colonize distant organs and form metastasis. Tumors develop their vasculature through a process called angiogenesis, from the Greek words *angeion*, vessel, and *genesis*, birth. This process is a hallmark of embryogenesis and tissue formation, while it does not take place in normal adult life, with the only exception of the female reproductive cycle. Cancer development, instead, entails the proliferation, migration, and remodeling of endothelial cells (the cell type that lines the vessel), resulting in the formation of new vessels. Cancer-associated angiogenesis can occur through different mechanisms [1]. The first to be identified, and probably the most common, is sprouting angiogenesis, namely the formation of new vessels from pre-existing ones. Intussusceptive angiogenesis, instead, consists of the formation of pillars across the lumen of a blood vessel, leading to the splitting into two new vessels. Vasculogenic mimicry relies on the ability of cancer cells to acquire endothelial-like properties (including certain phenotypic markers) and to form capillary-like vessels. Finally, recent observations have pointed to a specialized form of vasculogenic mimicry whereby endothelial cells (ECs) in tumor vessels derive for the transdifferentiation of cancer stem cells [2]. It should be mentioned that, under certain circumstances, some tumor types are also able to achieve their blood supply through a non-angiogenic mechanism that consists in “hijacking” the pre-existing vasculature, a process called vessel co-option [3].

The critical function of angiogenesis in the context of tumor growth and dissemination is mirrored by its clinical implications. Indeed, microvessel density, a direct function of tumor-induced vessel formation, has long been considered as an adverse prognostic factor [4], although it has become clear that in some cancer types a higher prognostic power can be achieved when dissecting tumor vasculature and quantifying, for example, immature vessels as opposed to all tumor-associated vessels [5].

Following the initial hypothesis that the induction of new vessels is a conditio sine qua non for tumor growth [6], several molecular mechanisms and players that drive or regulate cancer angiogenesis have been identified and characterized in detail. While the first angiogenic factor to be identified was basic fibroblast growth factor (bFGF) [7], the vascular endothelial growth factor (VEGF)/VEGFR system emerged as the most prominent signaling axis in cancer. Members of the VEGF family, indeed, exert a very potent angiogenic activity through their interaction with the VEGFR receptor tyrosine kinases on the EC surface, which triggers a cascade of signals that ultimately lead to angiogenesis [8]. Fibroblast growth factors (FGFs) represent another family of soluble angiogenic molecules which also act by stimulating high-affinity receptor tyrosine kinases, the FGFRs [9]. Of note, the VEGF and FGF systems have been shown to crosstalk with each other, possibly sustaining tumor angiogenesis in a synergistic manner [10,11,12]. Other signaling molecules that have emerged as major regulators of tumor vessels include the angiopoietins and their Tie-family receptors, ephrin/Eph and the Dll4/Notch system [13]. While the signal-receiving receptors are commonly expressed in ECs, their soluble ligands can be produced by cancer cells but also by other cell types, as part of the deregulated and aberrant microenvironment that results from the bidirectional crosstalk between the growing tumor and the host.

## 2. Antiangiogenic Therapy

Folkman’s hypothesis that tumors depend on the formation of new vessels for their growth beyond minimal size implied that preventing angiogenesis itself would be an efficacious antitumor strategy [6]. In principle, antiangiogenic treatments should offer tremendous advantages when compared to treatments directed against cancer cells, especially when besides preventing angiogenesis they also affect tumor vessels that are present already. First, the elimination of a single vessel would affect a high number of tumor cells that reside in the neighborhood and are supplied by that vessel. Second, ECs are generally considered to be genomically stable and, therefore, are not expected to develop drug resistance as the result of genome alterations. Third, drugs against cancer cells are normally unable to reach all areas of a tumor mass, due to the vascular barrier and the high interstitial fluid pressure, while the target of antiangiogenic compounds, the endothelial cell, is readily accessible from the circulation. Finally, as discussed below, tumor vessels are remarkably different from their normal counterpart, which implies that targeting their abnormal features would spare the vasculature of healthy tissues.

These considerations prompted many laboratories to design antiangiogenic strategies and to test their feasibility as anticancer treatments. The most prominent output of such a research effort has been the approval, in 2004, of bevacizumab, a VEGF-neutralizing monoclonal antibody, as the first antiangiogenic compound for tumor therapy [14]. Bevacizumab was initially approved for the treatment of metastatic colorectal cancer and, thereafter, for other tumor types including glioblastoma and lung, breast, and ovarian carcinoma. The approval of bevacizumab paved the way to a plethora of other compounds with antiangiogenic activity. The majority of them are directed against the VEGF/VEGFR signaling axis and include function-blocking antibodies and small molecule inhibitors [15]. More recently, promising results have been obtained with molecules that target other pro-angiogenic signals, such angiopoietins and Dll4, while other approaches are being investigated in many laboratories [16].

Most of the antiangiogenic approaches have proven very effective in preclinical models such as tumor-bearing mice. In spite of undoubted beneficial effects in several human neoplasms, however, it is fair to say that in most cases antiangiogenic therapy did not meet the expectations. Drugs interfering with VEGF function, for example, when used in combination with chemotherapy do increase progression-free and, to a lesser extent overall survival [17,18,19]. However, in the vast majority of clinical trials the survival benefit was quite modest and transient. Most tumors, indeed, are able to escape from VEGF blockades and to re-start growing, which implies the failure to fulfill the original objective of antiangiogenesis, namely keeping the tumor in a dormancy-like state [6]. Escape from VEGF-targeted therapies occurs through different mechanisms, including hypoxia-induced invasion and dissemination [20,21], stimulation of alternative angiogenic pathways such as FGF/FGFR and PDGF/PDGFR [22], recruitment of pro-angiogenic bone marrow-derived cells [23], but also through a shift towards angiogenesis-independent vascular supply such as vessel co-option [24,25] and vasculogenic mimicry [26,27,28].

Taken together, these findings point to the need, on one hand, for combinatorial therapies in order to interfere with the multiple mechanisms that tumors adopt to achieve blood supply and, on the other, for the definition of alternative vascularization pathways that may help to circumvent resistance to and evasion from current antiangiogenic treatments.

## 3. Vascular Normalization

**Figure 1:**
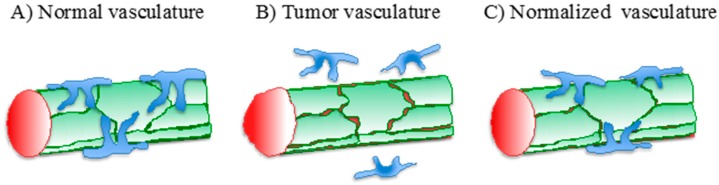
Aberrant tumor vessels and vascular normalization. (**A**) In normal vessels, endothelial cells (ECs) (green) are tightly adherent to each other, forming a continuous barrier. This, together with pericytes (blue), stabilizes the vessel wall; (**B**) In tumor vessels, ECs have an irregular morphology and are loosely attached to each other, resulting in intercellular gaps and, therefore, increased permeability; (**C**) Antiangiogenic treatments promote vascular normalization by restoring pericyte coverage and inter-endothelial adhesion, which revert vessel structure and function to a “normal-like” state. The red area indicates the vessel lumen.

The induction of deregulated vascular growth which sustains tumor expansion is accompanied by dramatic alterations in the structure and function of tumor-associated vessels. The latter, indeed, lack the typical hierarchical organization that characterizes the vascular tree in normal tissues and are often dilated and tortuous. At the ultrastructural level, ECs in tumor vessels exhibit a discontinuous and loosely attached basement membrane, loosened intercellular adhesion, and reduced coverage by mural pericytes (Figure 1B). These structural alterations have dramatic functional outcomes, which include vessel destabilization with reduced endothelial barrier function and increased vascular permeability, high interstitial fluid pressure, and poor perfusion of tumor tissue [29]. The resulting generation of a hypoxic environment triggers cancer cell escape through invasion and metastasis, thus favoring tumor progression.

The aberrant structure and function of tumor vessels also have crucial implications from a therapeutic standpoint. Indeed, the interstitial fluid pressure and the inefficient perfusion represent major obstacles to the delivery and the efficacy of systemically administered drugs. Furthermore, tumor hypoxia reduces the efficacy of various radio- and chemotherapy strategies which rely on the generation of radical oxygen species. Overall, these observations imply that restoring a normal-like vascular bed in tumors (i.e., vascular normalization; Figure 1C) would enhance their response to therapies.

In fact, genetically modified mouse models have provided insights into the molecular mechanisms that regulate tumor vascular normalization, and the latter has also been causally linked to improved efficacy of antitumor treatments [30,31,32,33,34,35]. In addition to genetic approaches, vascular normalization and improved drug response have also been achieved through the pharmacological inhibition of angiogenic pathways in various preclinical models [36,37,38,39]. Notably, recent studies have transferred this knowledge to patients and have shown that VEGF-targeted therapy can promote vessel normalization in human tumors [40] and impact the response to chemotherapy [41]. It is obvious that these findings impose a paradigm shift in the context of antiangiogenesis, since the original principles of “starving” the tumors by inhibition of new vessels and regression of the pre-existing ones need to be reconciled with the advantage of restoring a normally functioning vasculature in order both to reduce hypoxia and to favor delivery of anticancer drugs [17].

## 4. L1CAM

**Figure 2:**
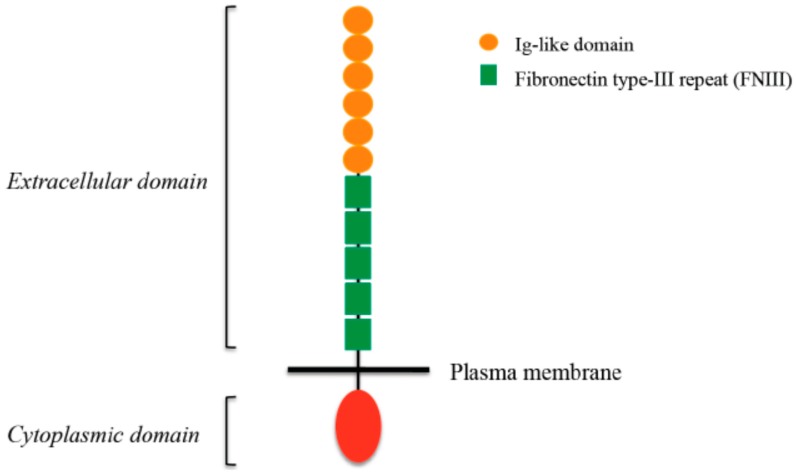
L1CAM structure. L1CAM is a cell adhesion molecule and is localized at the plasma membrane. It is composed by an extracellular portion that comprises six Ig-like domains (orange) and five fibronectin type III (FNIII) repeats (green), a short transmembrane domain, and a conserved cytoplasmic tail (red).

L1CAM (also known as L1 or CD171) belongs to the immunoglobulin superfamily of cell adhesion molecules (Ig-CAMs), a group of cell surface glycoproteins involved in intercellular recognition. It is composed of an extracellular portion with six Ig domains and five fibronectin type-III repeats, a transmembrane domain and a cytoplasmic tail (Figure 2).

Through its extracellular portion, L1CAM establishes homophilic interactions with other L1CAM molecules present on adjacent cells as well as heterophilic interactions with different partners, including other Ig-CAMs, integrins, receptor tyrosine kinases, and CD24. The cytoplasmic tail of L1CAM contains sequences that mediate its binding to ankyrins, which in turn interact with the spectrin-actin network [42,43]. Further anchorage to the cytoskeleton is provided by the binding of the RSLE amino acid motif to ezrin, a member of the ERM complex that is linked to actin [44]. The RSLE motif is also involved, via binding to the adapter protein AP2, in clathrin-mediated endocytosis of L1CAM [45].

**Figure 3:**
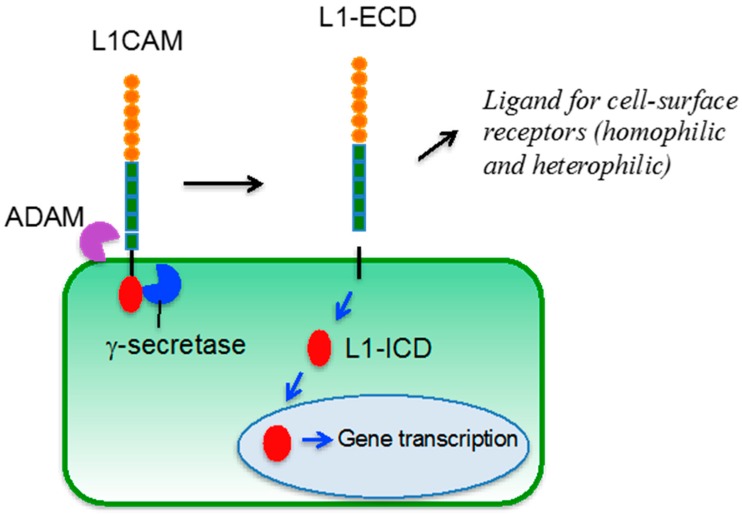
L1CAM processing at the plasma membrane. At the plasma membrane L1CAM undergoes proteolytic cleavage of the ectodomain mediated by a disintegrin and metalloproteinases (ADAM, purple object) which results in the shedding of a biologically active L1CAM extracellular domain (L1-ECD). Black arrows indicate the cleavage and release of L1-ECD. The membrane-associated portion becomes a substrate for γ-secretase (blue object) that cleaves the intra-membrane portion of L1CAM and induces the release of the intracellular domain of the protein (L1-ICD, red object). L1-ICD translocates into the nucleus and regulates gene transcription (blue arrows).

L1CAM can undergo membrane-proximal proteolytic cleavage mediated by members of the a disintegrin and metalloproteinases (ADAM) family, which induce shedding of the ectodomain that still retains biological activity [46]. The membrane bound C-terminal fragment is further processed by presenilin/γ-secretase activity [47], resulting in the release of an intracellular domain that is capable of translocating to the nucleus and regulating the expression of certain genes [48] (Figure 3).

The initial identification and characterization of L1CAM was carried out in the nervous system. Indeed, this Ig-CAM plays a central role in various neural processes, including axonal growth and pathfinding, neuronal migration, synaptic plasticity, and regeneration [49]. Indeed, mutations in the *L1CAM* gene cause a spectrum of neurological disorders that are collectively known as the L1 syndrome or CRASH syndrome [50], an acronym for Corpus callosum hypoplasia, Retardation, Adducted thumbs, Spasticity and Hydrocephalus.

The expression of L1CAM, however, is by no means restricted to the nervous system. Indeed, L1CAM has been found in specialized epithelia such as intestinal crypts [51], basal and intermediate layers of epidermis [52], renal collecting ducts [53], ovarian surface epithelium [54], as well as in various immune cell lineages [55,56].

The expression pattern of L1CAM is orchestrated by RE1-Silencing Transcription factor (REST, also known as neural restrictive silencer element, NRSE), a repressor which is low or absent in neurons and high in most non-neural tissues [57], as well as by other transcriptional and post-transcriptional mechanisms [58]. Interestingly, REST appears to modulate also the alternative splicing of L1CAM mRNA. Indeed, L1CAM occurs in two isoforms, the full-length variant which is typically expressed in neural cells, and the non-neural isoform in which the mini-exons 2 and 27 are spliced out. REST has been reported to modulate the expression of Nova2, a splicing factor that, in turn, governs the exon skipping in the L1CAM mRNA [59].

Finally, several studies have reported the aberrant expression of L1CAM in different tumor types [60]. In many cases, the expression level of L1CAM correlates with adverse prognosis [61]. For example, L1CAM has recently emerged as an important prognostic biomarker in endometrial carcinoma [62,63]. The association with poor outcome in patients is fully supported by the functional role of L1CAM in cancer cells, where it promotes epithelial-mesenchymal transition, motility, and invasion, as well as chemoresistance [64]. Such a role appears to be cell context-dependent: for example, L1CAM promotes cell-cell adhesion in normal ovarian surface epithelial cells while it stimulates cell motility in their transformed counterpart (i.e., ovarian carcinoma cells). Of note, L1CAM also contributes to the transendothelial migration of ovarian cancer cells [54]. Such a role of tumor-derived L1CAM in the crosstalk with vascular endothelium has been further investigated in breast cancer cells, where L1CAM mediates the interaction with the vasculature during metastatic dissemination to the brain [65] and to the lung [66]. Along this line, glioblastoma stem cells utilize L1CAM to bind α_v_β_3_ integrin in ECs and trigger angiogenesis-related events [67].

## 5. L1CAM in Tumor Vasculature

**Figure 4:**
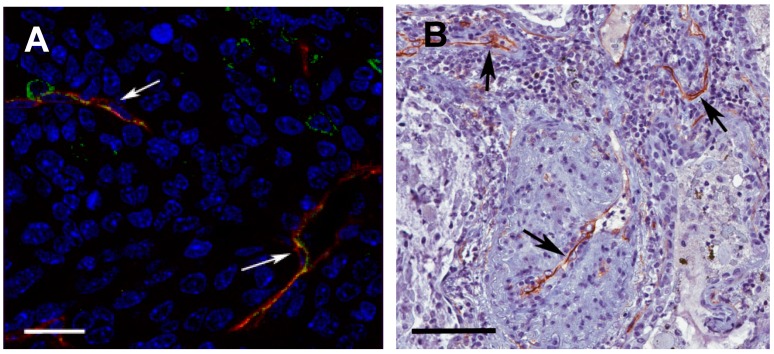
Expression of L1CAM in tumor vessels. (**A**) Mouse orthotopic, syngeneic model of pancreatic carcinoma (Panc02 cells transplanted into the head of the pancreas) co-stained for the vascular marker CD31 (red) and L1CAM (green). Scale bar, 15 μm; (**B**) Human lung carcinoma stained for L1CAM (brown). Arrows indicate L1CAM-positive tumor vessels. Scale bar, 150 μm. F.A. and U.C., unpublished data.

An unexpected finding that has received little attention until recently is the expression of L1CAM in the vasculature of various solid tumors, as exemplified in Figure 4.

By 1997, Felding-Haberman et al. had already reported de novo expression of L1CAM in angiogenic vessels associated to squamous cell carcinoma, but not in the quiescent vessel of healthy skin [68]. Thereafter, vascular expression of L1CAM was detected in melanoma [69], smooth muscle tumors [70], and neural tumors [71]. We have screened a wide variety of epithelial tumors and found L1CAM in the vasculature associated with carcinomas of the breast, ovary, prostate, colon, stomach, pancreas, thyroid, and lung [56,72]. The expression of L1CAM in the endothelium of pancreatic tumor vessels was also reported by Issa et al. [73].

Endothelial expression of L1CAM appears to be under the control of angiogenic and inflammatory cytokines that are abundantly released in the tumor microenvironment, including VEGF-A, angiopoietin-like 4, tumor necrosis factor-α, interferon-γ, and transforming growth factor β1 [56,73].

While the functional role of L1CAM in cancer vasculature has not been fully elucidated, the data available so far indicate a wide-spectrum function. Most of the studies have focused on the contribution of endothelial L1CAM to the interaction of cancer cells with the vessel wall. While, as discussed in the previous section, tumor cell-derived L1CAM mediates their binding to the vasculature [54,65,66,67], it is clear that a similar function is also carried out by L1CAM expressed in the vascular counterpart. In melanoma cells, for example, the α_v_β_3_ integrin establishes heterophilic interactions with L1CAM in the endothelium, thus enhancing the migration of tumor cells across the vascular barrier [74]. Neuropilin-1, a binding partner of L1CAM in the nervous system [75], was found in pancreatic carcinoma cells and proposed to interact with endothelial L1CAM, again favoring transendothelial migration [73]. In breast cancer cells, instead, L1CAM itself and the other Ig-CAM ALCAM were reported to establish homophilic and heterophilic interactions, respectively, with L1CAM in ECs, promoting the adhesion of tumor cells to the endothelium [76].

The functional implications of de novo expression of L1CAM in cancer-associated vessels are not limited to the tumor-endothelial crosstalk. Indeed, vascular L1CAM exerts a cell-autonomous role in ECs. By combining a mouse model of L1CAM-negative pancreatic carcinoma with the endothelial-specific ablation of L1CAM, we implicated vascular L1CAM in tumor angiogenesis and, as a consequence, in tumor growth and metastasis [72]. The ectopic expression of L1CAM in ECs resulted in cell proliferation, migration, and tube formation [72], in line with the EC-autonomous role of L1CAM. Consistent with our findings, antibody-mediated neutralization of L1CAM inhibited the migratory and remodeling capacity of tumor-derived ECs [73]. It should be mentioned that the pro-angiogenic function of the soluble L1CAM ectodomain or fragments thereof has been reported [77,78,79,80]. These studies highlighted the ability of extracellular L1CAM, which could derive either from neighbor cells (e.g., tumor cells) or from ECs themselves through proteolytic release (see above), to trigger signal transduction in ECs. We have complemented these observations by showing that endothelial L1CAM also exerts a prominent cell-autonomous role in the regulation of gene expression [72,81]. Unexpectedly, L1CAM was implicated in the transcription of almost 1000 genes in ECs, including genes known to play a key role in angiogenesis, such as *VEGFA*, *VEGFC*, and *DLL4* [72]. Furthermore, L1CAM induced de novo expression of mesenchymal genes which, together with the morphological and functional changes observed upon ectopic expression of L1CAM in ECs, indicated that this Ig-CAM promotes endothelial-to-mesenchymal transition [72], a process that contributes to cancer progression [82]. Pathway analysis revealed that L1CAM orchestrates a number of gene networks, in particular of the Signal Transducer and Activator of Transcription (STAT) family of transcription factors. Among these, the IL6/IL6R/JAK/STAT3 axis emerged as a prominent effector of L1CAM-induced EC activation [72,81].

Previous studies in non-endothelial cell types demonstrated that L1CAM induces the expression of the β_3_ integrin subunit [83,84]. The latter, especially in the context of the α_v_β_3_ integrin, plays an important role in EC pathophysiology, particularly in tumor angiogenesis [85]. Furthermore, as discussed earlier, α_v_β_3_ represents a preferential binding partner and effector of L1CAM [68,74,86]. Thus, it is tempting to speculate that the regulation of β_3_ integrin expression contributes significantly to the multiple roles of L1CAM in tumor vasculature.

Finally, the genetic manipulation of endothelial L1CAM in the context of a mouse tumor model revealed that L1CAM in tumor vessels prevents their morphological and functional maturation. Indeed, ablating L1CAM from the tumor vasculature restored endothelial polarity, basement membrane deposition, and pericyte coverage. At the functional level, these structural changes were mirrored by a dramatic decrease in tumor vessel permeability [72]. Taken together, these findings point to L1CAM inactivation as a novel strategy to achieve vascular normalization in tumors.

## 6. Therapeutic Perspectives

So far, the only clinical application of L1CAM in patients consisted in the use of a radioactively labeled antibody as an imaging tool to detect neuroblastoma, a study that pointed to L1CAM as a suitable target for radioimmunotherapy of that tumor type [87]. Numerous studies in preclinical models of different tumor types have indeed supported such a notion [88], particularly in ovarian cancer where L1CAM is often upregulated [89,90,91]. All these studies capitalized on the chimeric mouse/human monoclonal antibody chCE7, which is internalized by target cells [92].

Most of the experiments to assay for the antitumor efficacy of L1CAM-targeting strategies have been performed with function-blocking antibodies. As elegantly reviewed elsewhere [60,93], the use of anti-L1CAM antibodies that block its pro-malignant function, either alone or in combination with cytotoxic drugs, frequently resulted in dramatic inhibition of tumor growth and/or dissemination. Monoclonal antibodies that have been tested in preclinical cancer models include L1-11A, which showed antitumor effects in ovarian carcinoma [94] and melanoma [95]. Interestingly, the inhibitory effect of L1-11A on ovarian cancer cell growth synergized with radioimmunotherapy based on the ^67^Cu-labelled anti-L1CAM antibody chCE7 [96], thus implying the feasibility of combinatorial L1CAM-targeted treatments. The antibody A10-A3 was reported to block tumor growth in a mouse model of intrahepatic cholangiocarcinoma [97]. L1-9.3 is a monoclonal antibody that recognizes the first Ig domain of L1CAM. In its IgG2a version, L1-9.3 exerted a potent inhibitory effect in ovarian cancer xenograft models [98]. Table 1 summarizes the studies performed with anti-L1CAM monoclonal antibodies in preclinical settings. The data available so far also indicate an acceptable toxicity profile, although this aspect should be investigated in much greater detail in view of the possible clinical application. Of note, also the liposome encapsulation of anti-L1CAM siRNA provided a therapeutic tool that suppressed the growth and metastasis of prostate cancer [99].

Although the knowledge in this regard is quite limited, L1CAM represents a promising therapeutic target also in the context of tumor vasculature. Early in vitro studies have documented the ability of L1CAM-neutralizing antibodies to inhibit proliferation, invasion, and tube formation in ECs [77]. Furthermore, pre-incubating tumor-derived ECs with anti-L1CAM antibodies prevented the adhesion and the transendothelial migration of pancreatic cancer cells [73]. Thereafter, in vivo studies were conducted on an orthotopic model of pancreatic carcinoma treated with L1CAM-neutralizing antibodies. The latter significantly inhibited tumor-associated angiogenesis, resulting in reduced tumor growth, strengthening the rationale for testing L1CAM-targeting approaches as novel antiangiogenic treatments.

**Figure 5:**
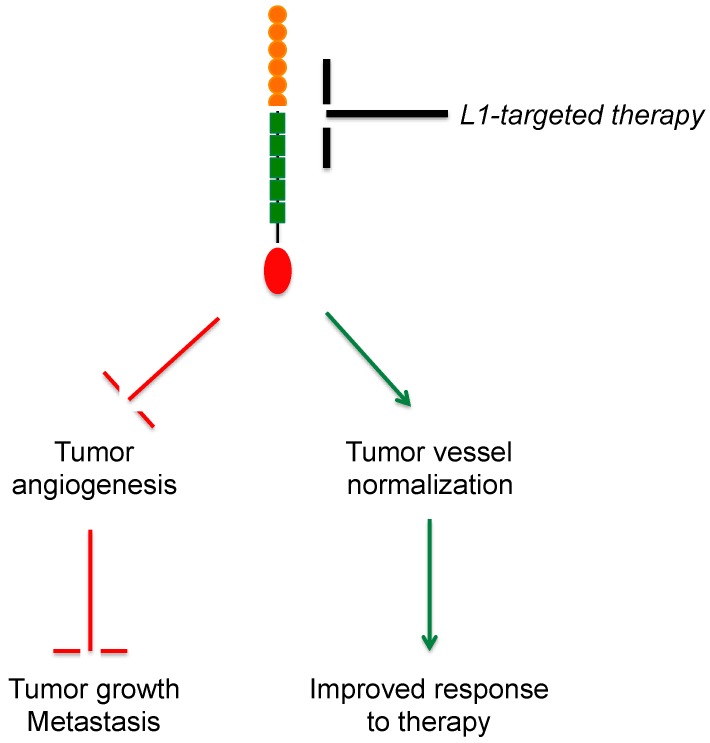
Outcome of L1CAM-targeted therapy in tumor vasculature. The inhibition of L1CAM function in tumor vessels (black T-shaped bar) is expected to result in decreased angiogenesis (top red T-shaped bar), thus leading to reduced tumor growth and metastasis (bottom red T-shaped bar), as well as to promote vascular normalization (top green arrow). The latter, in turn, would improve tumor perfusion and, therefore, delivery of systemically administered treatments, thereby enhancing the therapeutic response (bottom green arrow).

One of the most intriguing aspects of L1CAM biology is its ability to prevent vascular maturation (see previous section). The translational implications of this finding are supported by the observation that not only genetic ablation, but also antibody-mediated neutralization of L1CAM normalizes the tumor vasculature [72]. It remains unclear whether this effect depends on a true restoration of normal-like features (pericyte coverage, basement membrane, cell–cell junctions,) in previously aberrant tumor vessels rather than on the selective pruning of immature vessels, which leaves behind a more mature and functional vasculature. Regardless of the mechanism, it appears worthwhile to investigate in more detail and in additional preclinical models whether L1CAM-targeted therapies, possibly in combination with other antiangiogenic drugs, offer novel solutions for vessel-normalizing treatments. It would be of interest to elucidate whether L1CAM inhibition-mediated normalization improves tumor perfusion and, hence, delivery of cytotoxic drugs or other anticancer therapeutics (Figure 5).

In conclusion, compelling evidence supports the aberrant expression of L1CAM in cancer-associated vasculature, where it plays a pleiotropic role that ultimately sustains tumor angiogenesis and counteracts vessel maturation. While many aspects related to the pathobiology of L1CAM in the tumor vascular bed remain to be explored, this Ig-CAM may provide novel and alternative strategies in the context of vascular-targeting therapies in neoplastic diseases.

## Figures and Tables

**Table 1:** Use of anti-L1CAM antibodies in preclinical tumor models.

Antibody	Preclinical Model	Outcome	References
chCE7 (radioactively labelled)	Neuroblastoma; Ovarian cancer	High tumor uptake; Improved response to chemotherapy; Reduced tumor growth	[89,90,91]
A10-A3	Cholangiocarcinoma	Reduced tumor growth	[97]
L1-11A	Ovarian cancer; Melanoma	Inhibition of cancer cell invasion; Reduced tumor growth and dissemination	[94,95]
L1-9.3	Ovarian cancer	Reduced tumor growth; Macrophage infiltration	[98]
